# Hypercalcemia as a Cause of Kidney Failure: Case Report

**DOI:** 10.3889/oamjms.2016.044

**Published:** 2016-03-18

**Authors:** Olivera Stojceva-Taneva, Borjanka Taneva, Gjulsen Selim

**Affiliations:** 1 *University Clinic of Nephrology, Medical Faculty, Ss Cyril and Methodius University of Skopje, Skopje, Republic of Macedonia*; 2 *University Clinic of Cardiology, Medical Faculty, Ss Cyril and Methodius University of Skopje, Skopje, Republic of Macedonia*

**Keywords:** hypercalcemia, kidney failure, milk-alkali syndrome, metabolic alkalosis, case report

## Abstract

**BACKGROUND:**

Hypercalcemia is a common manifestation in clinical practice and occurs as a result of primary hyperparathyroidism, malignancy, milk-alkali syndrome, hyper or hypothyroidism, sarcoidosis and other known and unknown causes. Patients with milk-alkali syndrome typically are presented with renal failure, hypercalcemia, and metabolic alkalosis caused by the ingestion of calcium and absorbable alkali. This syndrome is caused by high intake of milk and sodium bicarbonate.

**CASE PRESENTATION:**

We present a 28-year old male admitted to hospital with a one-month history of nausea, vomiting, epigastric pain, increased blood pressure and worsening of renal function with hypercalcemia. His serum PTH level was almost undetectable; he had mild alkalosis, renal failure with eGFR of 42 ml/min, anemia, hypertension and abnormal ECG with shortened QT interval and ST elevation in V1-V4. He had a positive medical history for calcium-containing antacids intake and after ruling out primary hyperparathyroidism, malignancy, multiple myelomas, sarcoidosis, and thyroid dysfunction, it seemed plausible to diagnose him as having the milk-alkali syndrome.

**CONCLUSION:**

Although milk-alkali syndrome currently may be more probably a result of calcium and vitamin D intake in postmenopausal women, or in elderly men with reduced kidney function taking calcium-containing medications, one should not exclude the possibility of its appearance in younger patients taking calcium-containing medications and consider it a serious condition taking into account its possibility of inducing renal insufficiency.

## Introduction

Hypercalcemia is a common manifestation in clinical practice and occurs as a result of various underlying conditions. The reference range of serum calcium is maintained by a complex interplay of 3 major hormones, parathyroid hormone (PTH), 1,25- dihydroxy vitamin D (calcitriol) and calcitonin. They act primarily on bone, kidney and small intestine [1]. Primary hyperparathyroidism and malignancy account for approximately 80-90% of all hypercalcemic patients. Other causes of hypercalcemia include hypervitaminosis D, Milk-alkali syndrome, hyperthyroidism, hypothyroidism, Addison’s disease, sarcoidosis, thiazides, renal transplant or dialysis and unknown causes [2].

Patients with milk-alkali syndrome typically present with renal failure, hypercalcemia, and metabolic alkalosis caused by the ingestion of calcium and absorbable alkali. This syndrome is caused by high intake of milk and sodium bicarbonate, which was used for peptic ulcer treatment before the advent of histamine blockers and proton-pump inhibitors. Recently, use of calcium carbonate and vitamin D has increased among the elderly to prevent increases in osteoporosis, which in turn, may lead to hypercalcemia in the frame of milk-alkali syndrome [3].

We report the case of a man who presented with severe hypercalcemia, renal failure, and abdominal complaints.

## Case presentation

A 28-year-old male was admitted to hospital with a one-month history of nausea, vomiting, the epigastric pain increased blood pressure and worsening of renal function with hypercalcemia. He was diagnosed in the outpatient setting as having gastroesophageal reflux disease with biliary regurgitation and, therefore, was given high doses of calcium containing antacids. A therapy with calcium antagonists was also initiated because of increased levels of blood pressure.

Upon admission, he was well oriented, with slightly yellowish skin color, his blood pressure was 160/100 mmHg, his heart rate was 96/min. The rest of the physical examination was normal. He was a non-smoker and his past medical history was normal. The following initial analyzes have been performed:

**Table 1:** Pertinent laboratory values at admission

Test	Patient Values	Reference Ranges
Calcium	3.6	2.1-2.55 mmol/l
Ca^2+^	1.86	1.3-1.6 mmol/l
Albumin	41	35-50 g/l
Total protein	71	65-80 g/l
WBC count	10.2 × 10^9^/L	4-9 × 10^9^/L
Haemoglobin	8.7	14.0-18.0 g/dL
Haematocrit	0.28	0.37-0.54
ESR	38 mm/hr	4-10 mm/hr
Sodium	138 mmol/L	135-145 mmol/L
Potassium	3.4 mmol/L	3.8-5.5 mmol/L
Chloride	93 mmol/L	99-108 mmol/L
Bicarbonate	36 mmol/L	22-29 mmol/L
Blood urea nitrogen	8.2	3-7.8 mmol/l
Creatinine	233	45-109 μmol/L
Phosphate	0.9	0.8-1.4 mmol/L
Alkaline phosphatase	60 U/L	11-85 U/L
Free T4	18.03	8.41-17.65 pmol/L
PTH	2.4	15-65 pg/ml

Bilirubin (total)	25	6.8-20.5 μmol/L
Bilirubin indirect	19	5.1-13.6 μmol/L
Urinary excretion of Proteins	0.68	0.2 g/dU
Uric acid	539	150-450 μmol/L

**Figure 1:**
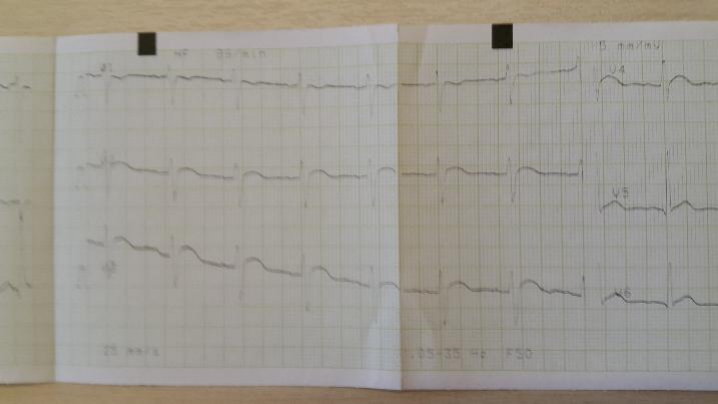
*ECG changes as a result of hypercalcaemia hypercalcaemia (V1-V3)*.

**Figure 2:**
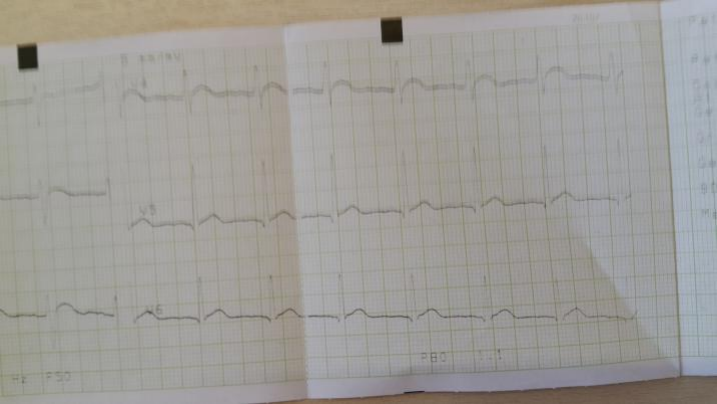
*ECG changes as a result of hypercalcaemia (V4-V6)*.

The patient had severe hypercalcemia and anemia, renal insufficiency, metabolic alkalosis. Serum parathyroid hormone was almost undetectable. His lipids were normal, too and urine culture negative. All the available tumor markers were normal (CEA, AFP, CA 19-9, NSE, CYFRA 21-1, PSA, Ferritin). He was hepatitis B, C and HIV negative. Renal ultrasound was normal. Ultrasound of the parathyroid glands was normal. Computerized tomography of the abdomen showed normal findings. Bone biopsy showed osteoporosis and scattered zones with osteonecrosis. Renal biopsy showed tubulointerstitial lesions with calcium deposits in the interstitial tissue. Chest X-ray was also normal. Endoscopy of the upper gastrointestinal tract while hospitalized showed no pathologic changes. Beta-2 microglobulin, acid phosphatase, kappa and lambda light chains were normal. Coombs test and immunoelectrophoresis of proteins were negative. The calculated creatinine clearance at admission was 42 ml/min. The whole body Tc^99m^ MDP bone scan was normal, as well as the parathyroid glands Tc^99m^ MIBI scan. Cranial X-ray, as well as radiographs of hands and feet, was normal. He had mild metabolic alkalosis, and his ECG showed signs of hypercalcemia (shortened QT interval of 0.32 sec and abnormal ST morphology in V2, V3 and V4) (Figs. 1 and 2).

The patient was hydrated with intravenous fluid, treated with bisphosphonates, corticosteroids and calcium antagonists for his elevated blood pressure. Antacids were stopped promptly after admission. He was also given vitamin B_12_ and folic acid upon the recommendation of a hematologist. The serum level of calcium decreased slowly to 2.6 mmol/l at the 43^rd^ day after admission, and serum creatinine decreased to 154 μmol/l (calculated creatinine clearance 63.5 ml/min).

After ruling out the differential diagnosis of multiple myelomas, other malignancies, primary hyperparathyroidism, hyperthyroidism or hypothyro-idism, it seemed plausible that the cause of hypercalcemia might be the ingestion of calcium containing antacids prescribed by his primary physician for his gastroesophageal reflux disease. He was discharged from hospital after 45 days of hospitalization.

## Discussion

Calcium is the main component of bone skeleton and serves as the intracellular and extracellular messenger in numerous cellular events such as neuronal network, immune response, muscle contraction, and hormone secretion. Less than 1% of body calcium is in the extracellular space, maintaining the extracellular calcium concentration within a narrow range and is very important for calcium homeostasis. Ten percent of the plasma calcium is in a complex with anions like phosphate, citrate, and sulfate and only half of plasma calcium is in its free form (ionized form, iCa^2+^) and tightly regulated by hormones like parathyroid hormone (PTH), 1,25-dihydroxyvitamin D_3_(1,25(OH)_2_D_3_), calcitonin, and calcium itself. The kidney, intestine, and bone are the main target organs of these regulators, and the kidney plays a key role in the fine regulation of calcium excretion [4].

Hypercalcemia is common in the clinical setting and occurs in conditions when the entry of calcium into the circulation is greater than the urine excretion or bone deposition, as is the case of accelerated bone resorption or excessive gastrointestinal absorption or decreased renal excretion. Primary hyperparathyroidism and malignancy are the most common causes of hypercalcemia [2]. Milk-alkali syndrome previously accounted for about 12% of cases and ranks the third among the causes of hypercalcemia in hospitalized patients [5]. But, after the advent of nonabsorbable antacids and later histamine-2 blockers, the incidence of this syndrome dropped to less than 1% of etiologies of hypercalcemia in the mid-seventies [6]. The pathophysiology of the milk-alkali syndrome is poorly understood, particularly because only some individuals are affected by excessive ingestion, but it has been suggested that they absorb more calcium than others. Patients who appear to be at high risk for milk-alkali syndrome include old age, volume depletion and medication that reduces glomerular filtration rate, such as angiotensin converting enzyme inhibitors, angiotensin receptor blockers or non-steroidal anti-inflammatory agents. Hypercalcemia causes reduced glomerular filtration rate, increased sodium excretion and depletion of total body water, leading to increased bicarbonate reabsorption and metabolic alkalosis. Alkalosis enhances calcium reabsorption in the distal nephron, thus, aggravating the hypercalcemia [7].

The modern version of the milk-alkali syndrome is now known as a calcium-alkali syndrome. It is the third most common cause of hypercalcemia in a retrospective study at a single center, where many patients reported consuming less than 2 g of elemental calcium per day in the form of calcium carbonate [8]. Whereas the traditional milk-alkali syndrome affected younger male patients with peptic ulcer disease, the demographics have changed to post-menopausal women, solid organ transplant recipients, pregnant women, bulimic patients, and those on dialysis [9].

Our patient had high levels of serum calcium, as in malignancies, thus our primary aim was to rule out malignant diseases. His PTH levels were suppressed and almost undetectable, thus in the differential diagnosis primary hyperparathyroidism was not to be considered. He also, had normal thyroid function, no signs of hypervitaminosis D, no medical history of medications that can cause hypercalcemia, neither signs nor symptoms of sarcoidosis. As he had positive medical history on calcium containing antacids intake because of his gastroesophageal reflux, while being volume depleted for his vomiting, and having already mild metabolic alkalosis, it was plausible to make an association of his medical history and the presence of the milk-alkali syndrome. The patient had ECG signs of hypercalcemia, shortened QT interval and elevated ST segment in V1-V4, mimicking STEMI (ST elevation myocardial infarction). These ECG abnormalities have been seen in other patients, too [10, 11]. He developed transitory renal insufficiency, with a complete remission few months after discharge. All signs and symptoms disappeared, his blood pressure returned to normal, and he has regular nephrology controls for 10 years now, with all his laboratory data being normal. He takes no medication and is in perfectly well condition.

In conclusion, although milk-alkali or calcium-alkali syndrome currently may be more probably a result of calcium and vitamin D intake in postmenopausal women, or in elderly men with reduced kidney function taking calcium-containing medications, one should not exclude the possibility of its appearance in younger patients taking calcium-containing medications and consider it a serious condition taking into account its possibility of inducing renal insufficiency.
